# The maternal and neonatal effects of adding tramadol to 2% lidocaine in epidural anesthesia for cesarean section

**DOI:** 10.5812/kowsar.22287523.1271

**Published:** 2011-07-01

**Authors:** Farnad Imani, Saeid Reza Entezary, Mahmoud Reza Alebouyeh, Suzan Parhizgar

**Affiliations:** 1 Department of Anesthesiology and Pain Medicine, Rasoul-Akram Medical Center, School of Medicine, Tehran University of Medical Sciences, Tehran, IR Iran; 2 Department of Anesthesiology, Texas Tech University, Health Science Center, Lubbock, Texas, USA

**Keywords:** Tramadol, Lidocaine, Epidural anesthesia, Cesarean section

## Abstract

**Background:**

Opioid analgesics are commonly added to epidural local anesthetics to improve analgesia during surgery.

**Objectives:**

The goal of this study was to evaluate the maternal and neonatal effects of adding different doses of tramadol to 2% lidocaine in the epidural anesthesia for cesarean section.

**Patients and Methods:**

Ninety pregnant patients who were candidates for cesarean section under epidural anesthesia were randomly categorized into three groups. Group L received 2% lidocaine. In the LT50 and LT100 groups, 50 and 100 mg of tramadol were added to epidural 2% lidocaine. For additional analgesia during surgery, 2% lidocaine through epidural catheter or IV sufentanil were administered. Analgesia after surgery was provided by IV injection of meperidine. Onset and duration of sensory and motor blockades, total drug consumption, neonatal Apgar score, and complications were recorded.

**Results:**

In the LT100 group, onset of complete sensory and motor blockade at T6 was less than in the two other groups, but the highest level of sensory blockade and two segment regression and duration of motor blockades between the LT50 and LT100 groups were not significantly different, although they were higher and more prolonged than in the L group. Average lidocaine and sufentanil consumption during surgery between the LT50 and LT100 groups were not significantly different but were lower than in the L group. The incidence of maternal complications and neonatal Apgar scores were not significantly different between the three groups. In the LT50 and LT100 groups, the time until the first request for analgesics after surgery was prolonged, and average meperidine consumption was less than in the L group.

**Conclusions:**

The addition of tramadol to epidural 2% lidocaine offers advantages in cesarean section.

## 1. Background

Epidural anesthesia is one of the most common methods used for anesthesia during cesarean section. The method’s advantages are reducing the maternal and neonatal side effects of intravenous (IV) or inhaled anesthetics, controlling the level of anesthesia compared with spinal block and lowering the incidence of hypotension and postdural puncture headache. Local anesthetics, such as lidocaine, have been used for this purpose; however, some disadvantages accompany epidural anesthesia with lidocaine, such as short duration of action and its effects on the neonatal Apgar score (1). Studies have examined whether adding various drugs to the epidural solution can decrease these unwanted effects (2-6). Among these, opioids have widely been used in various methods of epidural anesthesia. Tramadol is a synthetic µ-opioid agonist, has been used in different routes, such as intramuscular, intravenous, and oral, to reduce pain after the operation (7). Epidural tramadol as an adjuvant for local anesthetics during cesarean section has not been studied; however, it has been reported that the use of tramadol in the epidural route in other medical situations has satisfactory results (8).

## 2. Objectives

The purpose of this study was to evaluate the maternal and neonatal effects of adding tramadol (50 and 100 mg) to 2% epidural lidocaine in parturient women undergoing elective cesarean section.

## 3. Patients and Methods

After approval from the Medical Ethics’ Committee and having obtained written informed consent, 90 pregnant patients, ages 20 to 40 years, ASA physical status I or II, and undergoing elective term caesarean section were enrolled in this prospective and randomized study. The participants were randomly assigned to one of three groups (30 in each group) according to a random-number table. Exclusion criteria included ASA physical status higher than II, preexisting neurological or spinal disease, presence of factors threatening the neonate (e.g., placenta previa, placenta abruptio, preeclmpsia, fetal distress, or prematurity), alcohol or drug abuse, known hypersensitivity to local anesthetics or tramadol, vertebral column abnormalities (e.g., scoliosis), local infections, and patient refusal. Upon the participant’s arrival to the operating room, an 18-gauge IV catheter was inserted and rapidly infused with 500 mL Ringer solution. All vital signs (ECG, heart rate, oxygen saturation, and noninvasive blood pressure) were continuously monitored throughout the operation until the patient was discharged to the recovery room. Epidural anesthesia was initiated by an anesthesiologist blind to the patient group. Under asepsis and local anesthesia, epidural anesthesia was performed with the patient in the left lateral decubitus position using an 18-gauge epidural Tuohy needle at the L 3-4 or L 4-5 intervertebral space, and the epidural space was identified by a loss-of-resistance technique. After negative aspiration, a 3-mL test dose of 2% lidocaine with epinephrine 1:200,000 (xylocaine®; AstraZeneca, UK) was given. If after 5 min there was no evidence of intravascular or subarachnoid injection, the epidural solutions, which were prepared by another anesthesiologist not involved in the care of patients, were slowly injected into the epidural space over 30 seconds. These solutions contained 2% lidocaine (20 mL) with 1:200,000 epinephrine (xylocaine®) plus 2 mL of saline 0.9% in the control group (L group). In the LT50 group, 50 mg (1 mL) of preservative free tramadol hydrochloride (50 mg/mL) (tadol®; KRKA, Slovenia) plus 1 mL of saline 0.9% were added to 20 mL of 2% lidocaine solution. In the LT100 group, 100 mg (2 mL) of tramadol hydrochloride was added to 20 mL of 2% lidocaine solution. At the end of the injection, an epidural catheter was inserted into the epidural space. Immediately afterwards, the patients were placed in the supine horizontal position until the end of the study. Evaluations were conducted for sensory blockade by the pinprick method and for motor blockade with the modified Bromage scale (Table 1). Onset of anesthesia at T6, the highest level of sensory blockade, the onset of regression in the two dermatomes, the duration of complete motor blockade, and the sedation score (Table 2) were evaluated during the operation and in the recovery room. Sensory and motor blockade, sedation score, and the other parameters were recorded at 2-minute intervals from the induction of epidural anesthesia to delivery, and every 5 minutes thereafter. The condition of the neonate was assessed at the first and the fifth minutes using the Apgar score. Oxytocin was administered to all parturients as a 10-unit IV bolus, and afterwards, at a rate of 20 units/h after delivery. Hypotension (defined as systolic arterial blood pressure of less than 90 mmHg or a 30% decrease from baseline level) was managed with left uterine displacement, accelerating IV crystalloid solutions infusion, and IV boluses of 5 mg ephedrine, as required. Bradycardia (heart rate of less than 60 beats/min) was treated with 0.5 mg of IV atropine. Respiratory depression (respiratory rate of less than 6 beats/min or Spo2 less than 90%) was documented. Endotracheal intubation and general anesthesia were conducted when maternal apnea lasted longer than 20 seconds or enabled to speech or if the mother lost consciousness or did not respond to stimuli. If the Apgar score for the neonate was less than 7, neonatal resuscitations, such as oxygen by face mask, were performed. If the Apgar score was less than 5, endotracheal intubation was conducted afterwards. Patients complaining of nausea and vomiting received 10 mg of methoclopramide via IV. When the analgesia was not enough during the surgery or in the next hour in the recovery room, the mothers were given 2% lidocaine (5 mL) with 1:200,000 epinephrines via the epidural catheter. After 10 minutes, IV sufentanil (5 µg) was administered each time there was a need for more analgesia. Patients were transferred to the recovery room for an hour after surgery, and after removal of the catheter, the patients were transferred to the ward. If they had the visual analogue pain scores (VAPS, 0–10 scale: 0 = no pain, 10 = worst pain imaginable) of more than 3 in the ward, IV meperidine (20 mg) was administered. Moreover, the time of the first request for analgesics and the total dose of analgesics for the next 24 hours were recorded. Complications such as nausea and vomiting, bradycardia, hypotension, pruritus, and skin rash were noted. The information form included patient and surgical characteristics (including age, body weight, height, skin to uterus incision time, uterus incision to delivery time, duration of surgery), onset of anesthesia at the T6 level, highest anesthesia level, duration of complete sensory blockade (the onset of regression in two dermatomes) and motor blockade (the time required for motor blockade to decrease one score), maternal sedation score, neonatal Apgar score, the doses of IV sufentanil and epidural lidocaine used during surgery and also recovery, the time of the first analgesics request, total dose of meperidine in the next 24 hours, and any complications experienced. Statistical analyses were performed using SPSS 12 for Windows. Data are presented as means. Parametric data were analyzed with one-way analysis of variance or Chi-square tests as appropriate. When overall within-group effects were significant, pair-wise multiple comparisons of means testing (Tukey’s method) or Chi-square or Fisher’s exact test were performed as appropriate. p < 0.05 were considered statistically significant.

**Table 1.** Modified bromage scale

Score	Definition
**0**	No motor blockade
**1**	Inability to flex the hip joint
**2**	Inability to flex the knee joint
**3**	Inability to move the ankle or foot

**Table 2.** Sedation score

Score	Definition
**0**	Agitated
**1**	Awake and calm
**2**	Sleepy
**3**	Mild sedation (sleep awakened by voice)
**4**	Moderate sedation (sleep awakened by painful stimulus)
**5**	Deep sedation (sleep not awakened by any painful stimulus)

## 4. Results

Data on the individual characteristics of the participants reveal no significant differences among the three groups (Table 3). As shown in Table 4, the onset of complete sensory and motor blockades were more rapid in the LT100 group (14 ± 0.3, 20 ± 0.5 min) than in the LT50 group (16 ± 0.4, 23 ± 0.6 min) and in the L group (18 ± 0.5, 26 ± 0.7 min; p < 0.05). The highest level of sensory blockade, onset of regression in two dermatomes, and the duration of complete motor blockade were not significantly different between the LT100 and LT50 groups; however, they were higher and longer than in the L group (p < 0.05). The total lidocaine and sufentanil consumption during surgery were similar in the LT100 and LT50 groups but lower than in the L group. Although, no differences were observed in the rate of maternal complications or neonatal Apgar score among the three groups, sedation scores of 2 or 3 were more frequent in the LT50 and LT100 groups than in the L group. In both the LT100 and LT50 groups, the first analgesic request after surgery occurred later than in the L group, and the total meperidine consumption during the first 24 hours after surgery was lower in the LT100 and LT50 groups than in the L group. The incidence of complications was not statistically different between any of the groups (p = 0.48). Pruritus, skin rash, and respiratory depression were not observed in patients.

**Table 3.** Patient and surgical characteristics. Data are expressed as Means. No significant differences

	L group(n = 30)	LT50 group(n = 30)	LT100 group(n = 30)
**Age (y) (Mean ± SD) **	30 ± 2	28 ± 3	31 ± 3
**Weight (kg) (Mean ± SD) **	75 ± 12	72 ± 8	74 ± 10
**Height (cm) (Mean ± SD) **	162 ± 10	157 ± 6	159 ± 8
**Skin to uterus incision time (sec) (Mean ± SD) **	11 ± 3	9 ± 2	10 ± 4
**Uterus incision to fetus delivery time (sec) (Mean ± SD) **	95 ± 15	85 ± 12	90 ± 10
**Duration of operation (min) (Mean ± SD) **	55 ± 10	58 ± 7	56 ± 9

**Table 4.** Results of epidural blockade, consumed drugs, and maternal and neonatal data

	L group (n = 30)	LT50 group (n = 30)	LT100 group (n = 30)
**Onset of sensory blockade at T6^a^ (min) (Mean ± SD) **	18 ± 0.5	16 ± 0.4	14 ± 0.3
**Onset of motor blockade ^a^ (min) (Mean ± SD) **	26 ± 0.7	23 ± 0.6	20 ± 0.5
**Anesthesia level upper than T6 ^b^ (No.) **	21	27	28
**Sedation score of 2 or 3 ^b^ (No.) **	0	8	10
**Apgar score of 9 or 10 in the first min ^c^ (No.) **	24	25	24
**Apgar score of 10 in the fifth min ^c^ (No.) **	30	29	30
**Two dermatome regression ^b^ (min) (Mean ± SD) **	48.5 ± 9	70 ± 12	75.6 ± 14
**Duration of motor blockade ^b^ (min) (Mean ± SD) **	151 ± 17	171 ± 19	174 ± 22
**Intraoperative sufentanil ^b^ (µg) (Mean ± SD) **	8.7 ± 0.6	5.7 ± 0.5	5.2 ± 0.6
**Intraoperative lidocaine ^b^ (mg) (Mean ± SD) **	125 ± 16	114 ± 14	110 ± 12
**First postoperative analgesics request ^b^ (h) (Mean ± SD)**	4.3 ± 0.2	5 ± 0.2	5.4 ± 0.1
**Postoperative meperidine ^b^ (mg) (Mean ± SD) **	160 ± 10	130 ± 5	125 ± 5
**Maternal complications ^c^ (No.) **	9	12	14

^a^ significantly different among all three groups (P < 0.05)

^b^ significantly different between L and LT50 and LT100 groups (P < 0.05)

^c^ no significant differences

## 5. Discussion

According to the study, adding tramadol to lidocaine for the epidural anesthesia for caesarean section resulted in longer and more rapid sensory and motor blockade. Moreover, adding tramadol decreased the need for analgesics and local anesthetics during surgery, delayed the time of the first request for analgesics after operation, and decreased the total dose of analgesics, without any effects on maternal complications or the neonatal Apgar score. Tramadol is an opioid analgesic with central effects and is structurally similar to codeine and morphine. Like codeine, tramadol has a methyl group, which explains the weak tendency to opioid receptors (9). In contrast to codeine, tramadol has two enantiomers and its analgesic effectsoccurs indirectly via central inhibitory monoaminergic pathways.

(+) − tramadol causes inhibition of serotonin reuptake, and (−) − tramadol causes inhibition of epinephrine reuptake at the level of α-2 adrenergic, which accentuates the inhibition of pain conduction in the spinal cord (10). Synergistic and complementary effects of these two enantiomers can increase analgesic efficacy and compliance to the racemic component of the drug. The complex pharmacokinetic and pharmacodynamic characteristics of tramadol result from the differences in its concentration in plasma and in the target organ and also from the pharmacokinetic interaction between the two enantiomers and their metabolites. Tramadol’s analgesic potency is similar to meperidine and one tenth of the potency of morphine. The use of intramuscular (IM) tramadol as an analgesic in delivery showed that the analgesic effect of 100 mg of tramadol (IM) is similar to 75 mg of meperidine, but neonatal respiratory depression is more common in the meperidine group (11, 12). A previous study of IM tramadol measured the serum level of tramadol in mothers and neonates and evaluated neonatal Apgar scores; the results showed that approximately all of the injected drug reached the fetus because the placenta is very permeable to tramadol, but it was metabolized in the neonate’s liver to its nonstereoselective metabolite M1 (O-dimethyl tramadol hydrochloride) (13). In addition, Siddik and colleagues injected epidural tramadol after Caesarean section to patients who used epidural anesthesia during the operation. Tramadol (100 and 200 mg) was injected at the end of the operation through an epidural catheter and compared with the placebo group (14). Compared with the placebo group, there was a delay in the first request for analgesics in the tramadol groups and a decrease in the analgesics dose used in the 24 hours after the operation, but no difference in the two tramadol groups (100 and 200 mg). They concluded that 100 mg of tramadol after a Caesarean section can cause enough analgesia without respiratory complications in mothers. However, Baraka and colleagues compared 100 mg IV of tramadol with 100 µg IV of fentanyl and evaluated their effects on induction time in general anesthesia during Caesarean section and on PO2 and PCO2 of umbilical vein and neonatal Apgar scores (15). Tramadol caused a greater decrease in PO2 and a greater increase in PCO2 compared with fentanyl, but first- and fifth-minute Apgar scores were similar in the two groups. In another study by the same researchers, 100 mg of tramadol through the epidural route in patients with epidural anesthesia for major abdominal surgeries results in better analgesia and has fewer complications postoperation compared with 4 mg of morphine (16). Furthermore, Delilkan et al. showed that epidural injection of various doses of tramadol (50 and 100 mg) and 0.25% bupivacaine after surgery resulted in better analgesia and longer periods between epidural injections in the 100-mg tramadol group, although nausea and vomiting were also more common in this group (17). In addition to epidural injection of tramadol in humans, it has central-nervous blockade effects when given intrathecally in animals that are dose dependent (18). In addition to the analgesic effects of tramadol, researchers have also studied the drug’s anesthetic influences in vitro and found a local anesthetic effect (19). Another in vitro study, the amount of nervous conduction blockade of opioid local anesthetics was evaluated, and the results indicated that tramadol, although weaker than lidocaine, caused nervous conduction blockade with the same mechanism that may result from its interaction with calcium receptors (20). In another study by the same author, lidocaine in sodium channels blocks has been more effective than tramadol, but tramadol has been more effective than lidocaine on K channels blocks (21). The local anesthetic characteristic of tramadol can have an important role when used simultaneously with other anesthetic agents in anesthetic methods; for instance, adding tramadol to mepivacaine in axillary blockade results in improvement of pain after surgery (22). Also, in our previous study, injection of tramadol with lidocaine in supraclavicular brachial blockade accentuated sensory and motor blockades without any side effects (Imani F, Entezary SR, unpublished data). However, a potential concern is the probability of neurotoxicity followed by the direct use of tramadol on the nervous system, but in one study that applied tramadol directly to the sciatic nerve in rats, the drug had local anesthetic effects without any consequent motor dysfunction in the animal subjects (23). These findings indicate that tramadol is not neurotoxic when used beside the nerve sheet. The present study shows that adding tramadol to 2% lidocaine for epidural anesthesia in cesarean sections accentuates sensory and motor blockade effects without any increase in complications and can be appropriate in such surgeries.
